# Bupivacaine-Sufentanil Versus Bupivacaine-Fentanyl in Spinal Anesthesia of Patients Undergoing Lower Extremity Surgery

**DOI:** 10.5812/aapm.12091

**Published:** 2014-03-08

**Authors:** Valiollah Hassani, Gholamreza Movassaghi, Reza Safaian, Saeid Safari, Mohammad Mahdi Zamani, Maryam Hajiashrafi, Minow Sedaghat

**Affiliations:** 1 Minimally Invasive Surgery Research Center, Iran University of Medical Sciences, Tehran, Iran; 2 Department of Anesthesiology, Rasoul Akram Medical Center, Iran University of Medical Sciences, Tehran, Iran

**Keywords:** Analgesia, Anesthesia, Spinal, Bupivacaine, Fentanyl, Sufentanil

## Abstract

**Background:**

The addition of intrathecal opioids to local anesthetics seems to improve the quality of analgesia and prolong the duration of analgesia, when using a subarachnoid block in Iranian patients with their specific pain tolerance.

**Objectives:**

The aim of this study was to evaluate the effects of adding fentanyl or sufentanil, to intrathecal bupivacaine, in terms of the onset and duration of; sensory block, motor block, hemodynamic effects and postoperative pain relief.

**Patients and Methods:**

This randomized clinical trial included 90 patients who underwent orthopedic lower limb surgeries. Subjects were divided into experimental groups; intrathecal fentanyl 25 µg (F), and sufentanil 2.5 µg (S), along with a placebo 0.5 mL normal saline (C) group, which were added to bupivacaine 0.5%, 15 mg. Duration of complete and effective analgesia was recorded (by a visual analogue scale-VAS). The pain scores were assessed postoperatively. Intraoperative mean arterial pressure (MAP), heart rate and oxygen saturation (SPO^2^) were recorded. The incidence of side effects such as; nausea, vomiting, pruritus, shivering, bradycardia and hypotension were also recorded.

**Results:**

MAP and heart rate results showed no significant changes at the designated time points among the three groups (P > 0.05). However, SPO2 and VAS showed significant changes at the designated time points among the three groups (P < 0.05). The duration of complete and effective analgesia was also significantly longer in the sufentanil group (P < 0.05). Motor block did not exhibit any significant difference (P = 0.67). Only pruritus as a side effect was significantly higher in the sufentanil group (P < 0.05), while all other evaluated side effects were significantly lower in the sufentanil group (P < 0.05).

**Conclusions:**

The addition of 2.5-3 mcg sufentanil to 15 mg 0.05% bupivacaine maintained the patient’s hemodynamic stability similar to fentanyl. Intrathecal sufentanil added to bupivacaine,when compared with fentanyl, may lead to prolonged duration of analgesia, facilitate the spread of the sensory block, increase mean SPO2 levels, and reduce overall side effects.

## 1. Background

Spinal anesthesia (SA) is widely used in abdominal and lower extremity's surgeries due to its safety and simplicity, as well as the shorter time period that it needs to be completed (1, 2). Regarding high morbidity and increased risk of postoperative bleeding and nausea during the postoperative period in general anesthesia, SA seems to be a better method. Nowadays, the most common drug used for SA is bupivacaine (3). However, in addition to its inclusive effects, SA can result in urinary retention and prolong a patients' stay in the hospital. Furthermore, due to its short duration of action, bupivacaine needs other adjuvant drugs to increase its duration of action, such as opioids (4, 5). These drugs have a synergistic effect with bupivacaine and they can improve the anesthesia and prolong its activity (6). Using opioids results in a high incidence of nausea, urinary retention and respiratory depression (7). Fentanyl and sufentanil, which are lipophilic opioid drugs, have replaced other kinds of opioids, as they are highly soluble in lipids and potent blocking opium receptors, with fewer side effects (8). A meta-analysis, which investigated 28 randomized trials in 2013, indicated that the effects of intrathecal local anesthetics were only comparable with a reduced dose of the same local anesthetics when used with a concomitant opioid. In this meta-analysis, 19 trials used bupivacaine as local anesthetics, and 23 trials used fentanyl as a concomitant opioid. Sufentanil has been investigated in several studies and there was a greater requirement to clear its effects with intratechal anesthetic agents (9). In another meta-analysis of 65 trials conducted by Popping et al. and published between 1983 and 2010, included 3338 patients, of whom 1932 received opioids, it was found that morphine (0.05-2 mg) and fentanyl (10-50 μg) added to bupivacaine were the most frequently tested opioids and the authors demonstrated that there were not enough studies to allow for significant conclusions on the concomitant intratechal opioids; intrathecal buprenorphine, diamorphine, hydromorphone, meperidine, methadone, pentazocine, sufentanil, and tramadol (10). On the other hand, sufentanil reduces the bupivacaine dose required for desired anesthesia, and results in better cardiac output during surgery. The increasing demand for SA in orthopedic surgeries and the importance of the pharmacokinetic and pharmacology of new drugs made us appreciate the importance of studying the effects of new adjuvant drugs in SA (11, 12). Moreover, there are no studies about the specific aspects of this technique on different cultures with different pain experience; Dawson et al. demonstrated that middle eastern people are different in pain tolerance compared with European ones (13).

## 2. Objectives

We aimed to evaluate the improved effects of intrathecal sufentanil or fentanyl along with bupivacaine, during and after lower extremities orthopedic surgery.

## 3. Patients and Methods

This double-blind randomized clinical trial was conducted on elective patients who had orthopedic surgery on their lower extremities, and who were referred to the Rasoul-e-Akram and Firoozgar Hospitals, in 2012. Before any intervention, all potential candidates were informed about the study before they decided whether to participate or not, and a written consent was obtained. The patients were not charged any additional fees for the drugs at any stage of this study. All information was kept confidential and all authors were bound by the Helsinki declaration (IRCT: 201209045822N2).

Subjects who had indications for elective orthopedic surgery on their lower extremities and American Society of Anesthesiologists (ASA) level I or II, were entered into this study. Exclusion criteria were, history of discomfort from a previous SA, disability in the sitting position, coagulation disorders, non-compensated liver failure, severe renal failure (GFR < 60), heart failure (EF < 5%), any kind of heart block age, heart arrhythmia, confirmed hypertension, diabetes, obesity (BMI > 30), neurologic disorders, psychiatric disorders, history of spinal surgery, or any hypersensitivity to opioid or anesthesia drugs, pregnancy, alcohol or substance abuse. All patients who had any requirements for anesthesia higher than T4 or lower than T10 levels were also excluded. Subjects were randomly assigned to three equal groups; fentanyl, sufentanil or placebo group. Routine monitoring of electrocardiogram (ECG), pulse oximetry, and non-invasive blood pressure (NIBP), was conducted prior to the SA. All patients were routed with a green (18 gauge) catheter and infused with 3-4 cc/kg isotonic crystalloids. Subjects underwent intrathecal injection of 15 mg isobar bupivacaine, and one of the three following additives: 0.5-0.6 mL normal saline; 25- 30 mcg fentanyl or 2.5-3 mcg sufentanil. The syringes were filled with one of the above drugs by an anesthesiology technician and handed to the blinded anesthesiologist for injection. The intrathecal injection was carried out in the sitting position for all three groups. The checklists were filled by the same blinded anesthesiologist. Position of the patient was supine for all subjects, during the operation. The pneumatic cuff tourniquet was used for all patients with a cuff pressure of 300 mmHg, for the duration the surgery.

The patients received the spinal anesthetic through a 25-gauge Crawford needle. In the median approach the dural puncture was performed in the L3-4, or L4-5 interspaces, with the patients in the sitting position by an anesthesiologist, blinded to the syringes content. The anesthesia field was evaluated by a cotton peak (for heat perception), or a needle (for touching sense), every 15-20 seconds, then the motor block was evaluated using the Bromage scale as following: 0 = no paralysis; 1 = inability to raise extended leg; 2 = inability to flex knee; 3 = inability to move leg joints. Pulse rate, blood pressure and arterial blood oxygen saturation were evaluated every 2 minutes in the first 20 minutes, and every 5 minutes till the patient was transferred to the recovery room. The patient was excluded if any additional sedative or narcotic was used. The anesthesia start was calculated from the end of the injection and the anesthesia period was calculated from the start of the anesthesia till the first pain response of the patient after the end of surgery. The patient was asked about nausea, vomiting, pruritus and drowsiness. The patients were treated with 0.1 mg/kg metoclopramide if required. The patients' pain levels were evaluated in the 1st, 2nd, 6th, 12th, 18th and 24th hour after recovery by a visual analogue scale (VAS). Intravenous 0.5 mg/kg meperidine was administrated and repeated if the pain was not tolerated (VAS > 3). The time and dose were recorded. Any systolic blood pressure drop more than 20% or under 100 mmHg, and pulse rate drop to less than 60/min, were recorded during the surgery. Other demographic information, such as, sex, age, weight, height, physical examination, and medical history were obtained from the patients' medical files.

The data were evaluated and analyzed by SPSS (version 19) (SPSS Inc., Illinois, USA). All quantitative data were expressed as mean ± SD and qualitative data as No. (%). Repeated measurements analysis and post-hoc tests were used for comparison of SBP, DBP, PR and SPO_2_ during the surgery. P value less than 0.05 were considered significant.

## 4. Results

### 4.1. Demographic Data

Ninety patients were conveniently enrolled and divided into three equal groups. The mean age of the patients was 32 ± 15 years, and consisted of 23 females and 68 males (P = 0.35 and P = 0.66, respectively). Other demographic data are demonstrated in Table 1. None of the subjects required a general anesthesia during surgery due to pain intolerance and no one was excluded because of a high spinal block.

**Table 1.** Demographic Data and Adverse Effects of Three Groups of the Study ^a^

	Sufentanil	Fentanyl	Placebo	P Value
**Age, y**	35 ± 7	36 ± 11	41 ± 11	0.35
**BMI, kg/m²**	25 ± 1	25 ± 2	26 ± 2	0.59
**Surgery Duration, min**	154 ± 15	132 ± 41	98 ± 8	0.11
**Sex**	24	21	22	
Male				
Female	6	9	8	0.66
**ASA class **	22	19	25	
I				
II	8	11	5	0.21
**Adverse Effects, No.**				
Hypotension	5	8	6	0.62
Bradycardia	0	0	2	0.12
Nausea	0	4	1	0.01 ^b^
Vomiting	0	3	0	0 0.04 ^b^
Antiemetic Drug use	0	1	1	0.36
Pruritus	6	2	0	0.01 ^b^

^a^ Data are presented as mean ± SD

^b^ P value < 0.05

### 4.2. End Point Results

#### 4.2.1. Maximum Sensational Block

**Figure 1.**
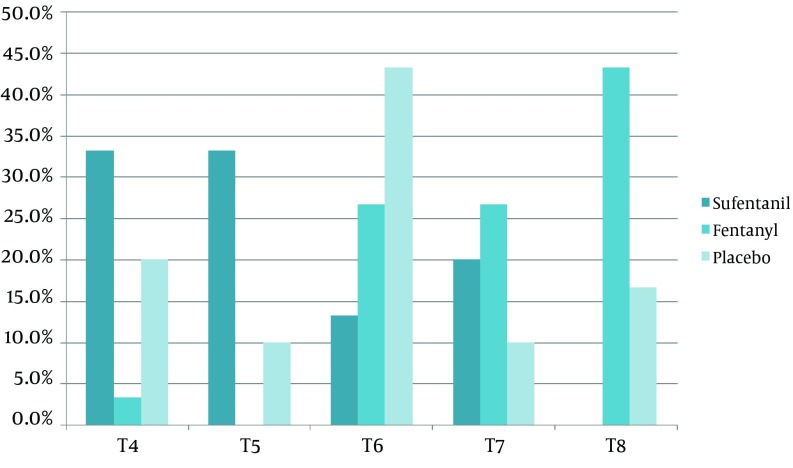
Percentage of Patients With Different Levels of Sensational Block (T4 to T8), in the Three Groups

The sensory block of the sufentanil group was more common at T4 and T5 levels, but sensory block of the fentanyl group was more common at the T8 level (P < 0.05). The maximum sensation level of block in all three groups is demonstrated in Figure 1. Significant differences were seen among the three groups (P < 0.05). The motor block did not exhibit any significant difference (P = 0.67).

#### 4.2.2. Vital Signs Changes

Hypotension, bradycardia, nausea, vomiting, metochloropramide requirement, pruritus, shivering and drowsiness were evaluated in all 3 groups and demonstrated in Table 1. As seen, both of nausea and vomiting were significantly lower in sufentanil group compare with fentanyl group (P < 0.05). Metoclopramide was administrated for 5 patients (1 subject in placebo group and 1 subject in fentanyl group) 

**Figure 2.**
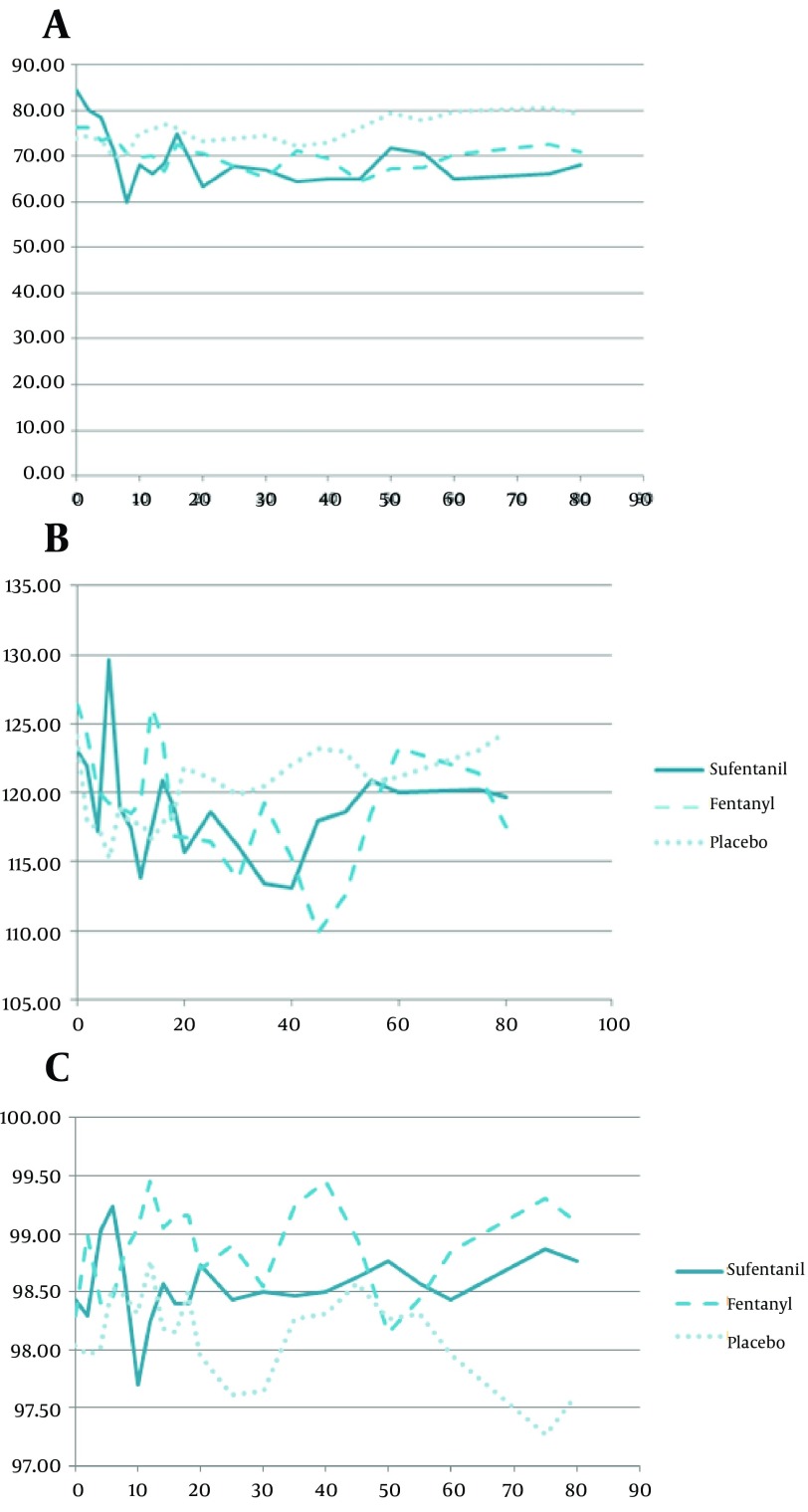
(a) Diastolic Blood Pressure (mmHg) and (b) Systolic Blood Pressure Changes (mmHg) and (c) SPO_2_ Changes (%), During Surgery (min) in Three Groups

Sufentanil had more stable blood pressures (the lowest changes) comparing with other groups (P < 0.05) (Figure 2 A, B). The HR changes did not have any significant difference among three groups (P = 0.99) which was 77 ± 13 per min, 78 ± 14 per min and 76 ± 11 in sufentanil, fentanyl and placebo groups respectively. However, SPO_2 _showed significant difference in Figure 2 C at 2ed, 4th, 6th, 10th and 12th minutes of evaluation, showing more stable SPO_2_ in sufentanil (P < 0.05).

#### 4.2.3. Pain Evaluation

**Figure 3.**
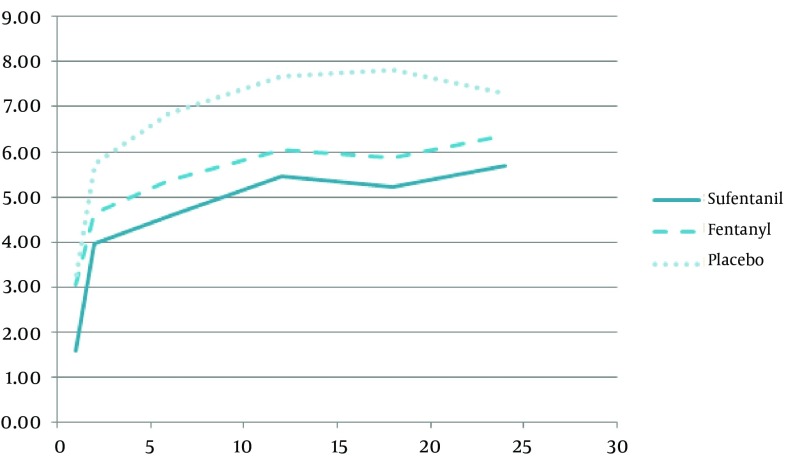
Visual Analogue Scale (VAS) of Postoperative Pain Changes (hour) in the Three Groups

Figure 3 presents the pain score which was in the lowest level for sufentanil group, and in the highest one in placebo group. The properties of intraoperative and postoperative analgesia are shown in Table 2. All VAS changes among three groups are significant (P < 0.05). True anesthesia time, effective Anastasia time and immobilization time were the highest in sufentanil group and additional opioid injection dose and time was the lowest in sufentanil group also.

**Table 2.** Properties of Intraoperative and Postoperative Analgesia in Three Groups ^a, b, c^

Time	Sufentanil	Fentanyl	Placebo	p^1^	p^2^	p^3^
**Analgesia time, min**	171 ± 19	168 ± 28	122 ± 20	0.0001	0.0001	0.542 ^b^
**Effective analgesia time, min**	214 ± 22	163 ± 21	146 ± 14	0.0001	0.012	0.0001
**Immobilization time, min**	226 ± 27	209 ± 14	159 ± 17	0.0001	0.0001	0.009
**First opioid request, hr**	4.9 ± 1.3	4.2 ± 2	3.8 ± 1.1	0.0001	0.156 ^b^	0.057 ^b^
**Dose of opioid, mg**	1 ± 0.6	1.1 ± 0.3	2 ± 0.6	0.0001	0.024	0.75 ^b^
**Pain Score, VAS**						
1st hour	1.6 ± 0.7	3 ± 1.2	3.3 ± 1	0.0001	0.341 ^b^	0.0001
2nd hour	3.9 ± 1.8	4.6 ± 1.6	5.7 ± 1.7	0.0001	0.0001	0.0001
6th Hour	4.5 ±2	5.4 ± 1.8	6.9 ± 2.7	0.0001	0.0001	0.004
12th hour	5.4 ± 2	5.5 ± 2.1	7.7 ± 3	0.0001	0.0001	0.033
18th hour	5.2 ± 1.8	5.9 ± 1.6	7.8 ± 1.9	0.0001	0.0001	0.0001
24th hour	5.7 ± 1.2	6.3 ± 1.9	7.3 ± 2	0.0001	0.003	0.012

^a^ data are presented as Mean ± SD.

^b^ VAS, Visual Analogue Scale; p^1^, Sufentanil vs. Placebo; p^2^, Fentanyl vs. Placebo; p^3^, Sufentanil vs. Fentanyl.

^c^ P value > 0.05.

#### 4.2.4. Side Effects

Hypotension was significantly lower, and pruritus had a higher prevalence rate in the sufentanil group (P < 0.05). Other side effects (bradycardia, nausea, vomiting and drowsiness) did not differ significantly (Table 1).

## 5. Discussion

Subarachnoid block or spinal anesthesia (SA) is a common method for abdominal and lower extremities' anesthesia. Many studies have demonstrated that SA can reduce venous thromboembolism in lower extremities' orthopedic surgeries and induce short term and rapid onset anesthesia (8, 14). Other adjuvant drugs can improve the anesthetic effects, such as opioids (15). Duration of anesthesia is prolonged by using opioids such as morphine; however, they may result in respiratory depression. Therefore, lipophilic opioids like fentanyl and sufentanil have been suggested (16).

Our study demonstrated significant changes in SPO2 and hemodynamic levels, among our three groups, and sufentanil had more stable vital signs than fentanyl or a placebo (P < 0.05), except for PR changes (P = 0.99). Blockage of preganglionic afferent sympathetic nerves at the level of T1-T4, results in a drop in blood pressure and a decrease in pulse rate, which can cause negative inotropic and chronotropic effects (17, 18). The SPO_2_ changes showed greater fluctuation than the other parameters, however, the sufentanil group had more stable SPO_2_ and none of the patients had a recorded SPO_2 _level of less than 96%, which were in accord with other studies results. Regarding assurance of results in the literature, respiratory depression is still one of the most annoying issues for anesthesiologists using lipophilic opioids. There is evidence which shows these drugs can also induce respiratory depression (19, 20).

Our study demonstrated no significant difference between the maximum levels of sensation block, which were at T4-T5 in the sufentanil group. Other studies had shown a level of T6 in both fentanyl and sufentanil (17, 21). Furthermore, sensed pain was significantly lower, and true and effective anesthesia were significantly higher in the sufentanil group, which supports the results of other studies (17). The µ agonists of both fentanyl and sufentanil induce lower voltage at the Ca^2+^ gates and open the K^+^ gates, which can drive the nerve into a post-synaptic hyper-polarization and result in reduced nerve conduction. Bupivacaine can inhibit the Na^+^ gates and help both fentanyl and sufentanil in anesthesia. Other studies have reported a 0 to 100% incidence of pruritus using lipophilic opioids, and we found that pruritus occurred in 20% of patients in sufentanil group, but this was the only side effects which had a higher prevalence among the three group. In other studies, nausea and vomiting had a dose dependent incidence of nearly 30%, although this study presented with a 0% incidence of nausea and vomiting using 2.5-3 mcg intrathecal sufentanil. However, these studies have also stated that the side effects may differ from hospital to hospital based on other factors, such as room temperature and the type of crystalloid used (22-24).

In summary, the addition of 2.5-3 mcg sufentanil to 15 mg 0.05% bupivacaine maintains patient’s hemodynamic stability, along with maximum sensation block and anesthesia duration. In addition, it can reduce the incidence of hypotension compared with fentanyl. Pruritus is more common with sufentanil than with intratechal fentanyl. Finally, sufentanil compared with other regular and lipophilic opioids is recommended for SA as an adjuant intratechal drug along with isobar bupivacaine.
